# The diversity of terrestrial arthropods in Canada

**DOI:** 10.3897/zookeys.819.31947

**Published:** 2019-01-24

**Authors:** David W. Langor

**Affiliations:** 1 Natural Resources Canada, Canadian Forest Service, 5320 – 122 St. NW, Edmonton, Alberta, T6H 3S5, Canada Natural Resources Canada, Canadian Forest Service Edmonton Canada

**Keywords:** Arachnida, biodiversity assessment, Biota of Canada, checklists, Entognatha, Hexapoda, Insecta, Myriapoda, surveys, taxonomy, Zygentoma

## Abstract

Based on data presented in 29 papers published in the *Biota of Canada* Special Issue of ZooKeys and data provided herein about Zygentoma, more than 44,100 described species of terrestrial arthropods (Arachnida, Myriapoda, Insecta, Entognatha) are now known from Canada. This represents more than a 34% increase in the number of described species reported 40 years ago (Danks 1979a). The most speciose groups are Diptera (9620 spp.), Hymenoptera (8757), and Coleoptera (8302). Less than 5% of the fauna has a natural Holarctic distribution and an additional 5.1% are non-native species. A conservatively estimated 27,000–42,600 additional species are expected to be eventually discovered in Canada, meaning that the total national species richness is ca. 71,100–86,700 and that currently 51–62% of the fauna is known. Of the most diverse groups, those that are least known, in terms of percent of the Canadian fauna that is documented, are Acari (31%), Thysanoptera (37%), Hymenoptera (46%), and Diptera (32–65%). All groups but Pauropoda have DNA barcodes based on Canadian material. More than 75,600 Barcode Index Numbers have been assigned to Canadian terrestrial arthropods, 63.5% of which are Diptera and Hymenoptera. Much work remains before the Canadian fauna is fully documented, and this will require decades to achieve. In particular, greater and more strategic investment in surveys and taxonomy (including DNA barcoding) is needed to adequately document the fauna.

## Introduction

During the last glacial maximum, approximately 21,400 years ago, most of Canada was covered by ice sheets. Small glacial refugia that existed in Beringia and on offshore islands (Roberts and Hamann 2015), in the Cypress Hills of Alberta and Saskatchewan (Pielou 1991), and possibly in what is now northern British Columbia (Marr et al. 2008), contained some species that persisted during glaciation, but the vast majority of the species now living on land and in freshwater in Canada recolonized the area from the un-glaciated south (Matthews 1979). In addition, thousands of non-native species, mainly from Europe and Asia, were later unintentionally or intentionally introduced to North America subsequent to European colonization and are now established in Canada (e.g., Langor et al. 2009, 2014, Klimaszewski et al. 2010; D Langor unpubl. data). Thus, most habitats of Canada are relatively new and most of the resident biota emigrated from the south under warming post-glacial conditions, so there has been little time for speciation to occur in Canada. Given this biotic colonization history, the overall diversity of species is much lower in Canada than further south (Danks and Smith 2017). Nonetheless, the diversity of species that reside in Canada is impressive, especially for terrestrial arthropods.

Herein, the grouping ‘terrestrial arthropods’ includes the subphylum Hexapoda (insects and relatives), the class Arachnida (spiders, mites, and relatives), and the subphylum Myriapoda (centipedes, millipedes, and relatives). Together these groups account for approximately 53% of the known species in Canada (excluding viruses), including estuarine and marine habitats (Table 1; Mosquin et al. 1995, CESCC 2016). Terrestrial arthropods are present in all parts of Canada’s landmass, except that which is permanently covered by snow and ice, and typically dominate in terms of species and trophic diversity. Despite their prevalence and importance, as a group, terrestrial arthropods are poorly known in Canada in terms of diversity and distribution, and detailed biology is known for only a tiny portion of the fauna.

The publication of *Canada and its insect fauna* (Danks 1979a) was a landmark for biodiversity science in Canada as it represented the first attempt to synthesize the state of knowledge about insect and other terrestrial arthropod diversity, distribution, and habitats in the country. Building in part upon Munroe (1956), Hugh Danks and his team of 60 authors synthesized a remarkable breadth and depth of knowledge in their review of: patterns of regional diversity in Canada; environmental and geological determinants of terrestrial arthropod habitats; and the diversity, distribution, and biology of each major terrestrial arthropod group in the country. Also remarkable is the fact that this work was accomplished without the benefits of personal computers, the Internet, and email.

The monograph, *Canada and its insect fauna*, has been highly influential and valuable to subsequent generations of scientists interested in the diversity of terrestrial arthropods (and biodiversity in general) in Canada. While the work still has enormous value, some parts, especially the chapters treating individual faunal groups, require updating as there has been enormous progress in the documentation of the Canadian fauna during the last 40 years. To address this need, in 2016, the Biological Survey of Canada (www.biologicalsurvey.ca) initiated a project to update the individual faunal chapters. This resulted in 29 papers that comprise the bulk of this Special Issue of ZooKeys, titled *The Biota of Canada – A Biodiversity Assessment. Part 1: The Terrestrial Arthropods* (Langor and Sheffield 2019), and those papers cover all terrestrial arthropod groups except the order Zygentoma (formerly called Thysanura). Zygentoma was not treated in a separate Biota of Canada paper because no authority could be found to lead it; however, the diversity of this order is briefly reviewed in Appendix I. Herein, I summarize and integrate the information and data presented in the Biota of Canada papers, highlight trends and patterns, and provide a future outlook.

## Species diversity and faunal affinity

### Current diversity

Authors of all Biota of Canada papers were asked to provide data that were current at time of writing (late 2017 to 2018) rather than relying solely on the most recent published checklist. The sources of these data are included in individual papers. The total numbers of species currently known from Canada for each group (Table 1) were extracted from those papers, with the exception of the Zygentoma (Appendix I). For all groups, only the number of described species (not subspecies) currently recorded from Canada are included in the tally. These totals were reported by family in each individual paper but were totalled mainly at the order and higher levels herein (Table 1), with the exceptions that the former orders Psocoptera and Phthiraptera are now considered to be part of the Order Psocodea (see discussion in Anonby 2019).

The total number of described species currently recorded from Canada is 44,103 with 88.3% represented by insects, 10.3% by arachnids, 1.1% by entognathous hexapods, and 0.3% by myriapods (Table 1). Remarkably, the proportional representation by these four faunal groups in Canada is similar to that for the global terrestrial arthropod fauna of ca. 1.2 M described species (global calculations based on data from Zhang (2011, 2013), from references provided within the other papers in the Biota of Canada issue, and from many other sources). For terrestrial arthropods, the described Canadian fauna represents 3.7% of the known global fauna. The six most diverse terrestrial arthropod groups in Canada are Diptera (21.8% of total Canadian fauna), Hymenoptera (19.9%), Coleoptera (18.8%), Lepidoptera (12.4%), Hemiptera (9.1%), and Acari (6.8%).

### Change since 1979

Before comparing current described species diversity (Table 1) to that reported in Danks (1979b), it was necessary to adjust the earlier reported species numbers to correct some errors and inconsistencies that have recently come to light (see footnotes of Table 1 for the details of adjustments). Danks (1979b) reported 33,577 terrestrial arthropod species from Canada, excluding Tardigrada, Pentastomida, and terrestrial Crustacea, which were covered in the earlier work but not herein. The adjusted total of 32,850 described species known in 1979 (Table 1) is still somewhat inflated as the 1915 species of Acari reported then (Lindquist et al. 1979) included both described and undescribed species, but it is not possible to now adjust this number downward to accurately reflect only described species known at the time (see Beaulieu et al. 2019).

The number of described terrestrial arthropod species in Canada is now at least 11,250 (34.3%) more than that known in 1979. The groups with the greatest growth in number of described species are Hymenoptera (2729 spp.), Diptera (2564), Coleoptera (1560), Lepidoptera (1348), Acari (>1082), and Hemiptera (932). In terms of proportional growth in described species, the groups (all of them with relatively low diversity) showing the largest increases are Pauropoda (no species reported in 1979, currently 23 spp.), Pseudoscorpiones (500% increase, currently 25 spp.), Strepsiptera (333%, 27 spp.), Diplura (300%, 6 spp.), Protura (300%, 9 spp.), and Solifugae (300%, 3 spp.). Most of the groups showing little or no increase in described species during the last 40 years are small groups (<25 species in Canada); however, notably the Siphonaptera (154 spp. currently known from Canada) show only a 2.0% increase in described species, despite considerable work on this group during the last 40 years, indicating that the fauna was already well documented by 1979 (Galloway 2019b).

### Proportion of North American fauna in Canada

Approximately 37% of the described terrestrial arthropod fauna of North America north of Mexico occurs in Canada (estimate is based on data extracted from sources used in papers in Langor and Sheffield (2019), from authors directly via personal communications, and from other sources). The groups with the highest proportion of the known North American fauna present in Canada are: Ephemeroptera (51.1%; Jacobus 2019), Siphonaptera (50.8%; Galloway 2019b), Hymenoptera – Ichneumonidae + Braconidae + Chalcidoidea (48.3%; A Bennett pers. comm.), Odonata (45.8%; R Cannings 2019 pers. comm.), and Diptera (43.3%; Savage et al. 2019; A Borkent pers. comm.). Groups that have predominantly southern distributions in North America and have a low percentage of their fauna present in Canada include Scorpiones (1.1%; http://www.angelfire.com/tx4/scorpiones/states.html), Solifugae (1.5%; Cushing and Brookhart 2019), Diplura (3.5%; Sikes 2019), Diplopoda (4.4%; Langor et al. 2019), and Pseudoscorpiones (4.8%; Cameron and Buddle 2019).

### Holarctic species

The large majority of the Canadian fauna is restricted to the Nearctic; however, there is also a significant proportion that has a naturally Holarctic distribution. While the Holarctic component cannot be readily calculated for the entire fauna, it is relatively well known for some large groups: Lepidoptera – 4.8% (Pohl et al. 2019), Coleoptera – 5.1% (Bousquet et al. 2013), Hemiptera – 4.3% (E Maw pers. comm.), and Ichneumonoidea (Hymenoptera) – <5% (A Bennett pers. comm.). The Holarctic portion of the fauna is remarkably similar among these four highly diverse groups. In contrast, 21.9% of the 471 species of Collembola known from Canada have Holarctic distributions although it is not currently possible to discern what proportion of those are non-native versus naturally Holarctic (M Turnbull pers. comm.). Overall, we estimate that <5% of the total native Canadian terrestrial arthropod fauna is Holarctic. As more taxonomic revisions are undertaken that consider the Holarctic fauna, it will likely be discovered that for many groups some putative Nearctic species are synonymous with species described in the Palaearctic, thereby increasing the number of Holarctic species. Furthermore, other species currently considered Holarctic will likely to be discovered to be sibling species, one Palaearctic and one Nearctic, thereby decreasing the number of Holarctic species. Thus, the actual proportion of the fauna that is naturally Holarctic is likely slightly different from that currently estimated.

### Non-native species

Many non-native terrestrial arthropod species have been introduced to Canada since the time of European colonization, most of them inadvertently and some intentionally, e.g., for biocontrol (Langor et al. 2009). The authors of most Biota of Canada papers considered and reported on the proportion of the fauna that is non-native. Two notable exceptions are the Diptera and the Hymenoptera. For these orders, the tally of known non-native species is based on information gathered from published literature and from consultation of taxonomists (D Langor unpubl. data); however, this database has not been updated since 2015 so it is likely that the numbers of non-native species are slightly under-estimated. In total, 2064 non-native terrestrial arthropod species (excluding Entognatha and Acari) are known from Canada and this represents ca. 5.1% of the total fauna (Table 1). The groups with the most non-native species are Coleoptera (639 spp.), Hemiptera (405 spp.), Hymenoptera (402 spp.), Lepidoptera (207 spp.), and Diptera (147 spp.). The groups with the highest proportion of their described fauna that is non-native are Zygentoma (100%), Dermaptera (67%), Mantodea (67%), and Blattodea (50%); these are mostly groups (with the exception of Mantodea) that have strong association with human dwellings, and many species have been transported around the world by human activities. For two groups, Acari and Entognatha, it is not yet possible to obtain good estimates of numbers of non-native species in Canada. The distribution of Entognatha (primarily soil-dwelling species and dominated by Collembola) is poorly understood and there are still serious gaps in taxonomy and distribution that prevent a meaningful assessment of which of the apparent Holarctic species are naturally Holarctic versus introduced. In the case of Acari, most groups are so poorly known in terms of taxonomy, distribution, biology, and phylogeny, that a meaningful analysis of geographic affinity cannot yet be undertaken (Beaulieu et al. 2019). The number of non-native parasitic Hymenoptera species is likely greatly underestimated, especially those that were intentionally introduced for the purposes of biocontrol as historical records are not complete (A. Bennett et al. 2019a). For most groups of terrestrial arthropods it is likely that additional non-native species already occur in Canada or may soon spread to Canada from established populations in the northern contiguous USA. Undoubtedly some species currently believed to be non-native may prove to have natural Holarctic distributions; however, it is conceivable that more than 2500 non-native terrestrial arthropod species are already established in Canada.

## DNA barcodes

One of the most significant scientific developments in biodiversity science since 1979, that is now greatly helping the process of documenting Canada’s (and the world’s) biota, is the use of DNA characters. Thus, in the current assessment of Canada’s terrestrial arthropod diversity, genetic data have been reported and used to estimate species diversity (Table 1). For animals, the utility of mitochondrial DNA, particularly the COI region (cytochrome c oxidase subunit I) as a source of taxonomically-relevant characters was already well recognized and exploited by the mid-1990s (e.g., Langor and Sperling 1995, Sperling and Hickey 1995). Folmer et al. (1994) first called attention to a 658-bp region of COI that had high phylogenetic value for resolving species and higher taxonomic levels across a wide variety of metazoan invertebrates, and they developed a set of universal primers for polymerase chain reaction amplification of the region. This so-called ‘Folmer region’ was later promoted in the ‘DNA barcode’ concept of Hebert et al. (2003) and is now the focus of most global barcoding efforts for metazoan animals. Thus, the advent of DNA barcoding and the enormous strides in genomics methods and data management and analyses has greatly enhanced the collection and utilization of genetic data for the purposes of taxonomy, diagnostics, and phylogenetics (Wilson et al. 2017). Due to the rapidly increasing availability of molecular data, especially in the barcode region, taxonomic and phylogenetics publications are increasingly integrating both morphological and molecular data to address questions. As of November 2018, the Barcode of Life Data System (BOLD; Ratnasingham and Hebert 2007) contains more than 6.6 million barcodes worldwide and 1.9 million are from terrestrial arthropods collected in Canada (A Telfer pers. comm.).

An algorithm was developed to group DNA barcodes with high similarity into clusters, forming Operational Taxonomic Units that are assigned unique and persistent Barcode Index Numbers (BINs; Ratnasingham and Hebert 2013). BINs have high concordance with species in most groups (Ratnasingham and Hebert 2013), although amongst terrestrial arthropods the standard barcode region is sub-optimal for species resolution in Odonata (Rach et al. 2017) so BINs have relatively low concordance with odonate species in Canada (Cannings 2019). In other groups as well, there are cases where valid species share BINs and other cases where a single species may have several BINs (e.g., Foottit et al. 2008, Davis and Landry 2012, Prous et al. 2017, Zahiri et al. 2017). Nonetheless, BINs are generally highly representative of species diversity: for example, in Lepidoptera, BINs have ca. 93% congruence with named species (deWaard et al. 2011, Zahiri et al. 2017), in Quediina (Coleoptera: Staphylinidae) there was 92% congruence between BINs and named species (Brunke et al. 2019), and in AraneaeBINs were able to discriminate 98% of 1018 Canadian species (Blagoev et al. 2016), which indicates the high value of DNA barcodes for providing taxonomic resolution.

More than 75,000 BINs have been assigned to terrestrial arthropod specimens from Canada, 86% of which are from insects (Table 2). The groups with the highest number of BINs assigned are Diptera (39.1% of total), Hymenoptera (24.4%), Acari (9.9%), Lepidoptera (7.7%), and Coleoptera (7.6%). At the other extreme, there are no barcodes from Canadian specimens of Pauropoda despite 23 known species from the country (Langor et al. 2019). The vast majority of BINs from terrestrial arthropods have family-level assignments. Of 1037 families with described species in Canada, 81% have associated BINs based on Canadian material (Table 2). Almost all families lacking BIN data from Canada are represented by 1–5 species and are uncommonly encountered. The percent of families with assigned BINs is highly variable across groups; the lower end of the range includes Pauropoda (0%), Siphonaptera (43%), Phthiraptera (47%), Diplura (50%), and Protura (50%). Groups with ≥10 families that have a high percentage of families with assigned BINs include Psocoptera (100%), Lepidoptera (95%), Diptera (94%), Trichoptera (92%), Araneae (91%), and Odonata (90%).

**Table 1.** Diversity of terrestrial arthropods in Canada.

Taxon	Adjusted no. described species known in Canada in 1979	No. species currently known in Canada	Percent change since 1979	Percent (no.) non-native species	Est. no. undescribed or unrecorded species in Canada	Percent of Canadian fauna known	Information sources
**Class Arachnida**							
Order Araneae	1249^1^	1477	18.3%	5.5% (81)	300–350	81–83%	R Bennett et al. 2019b; R Bennett pers. comm.
Order Opiliones	47	43	-8.5%	16.3% (7)	22	66%	Shultz 2019
Order Pseudoscorpiones	5	25	500.0%	4.0% (1)	27	48%	Cameron and Buddle 2019
Order Scorpiones	1	1	0	0	0	100%	Cameron and Buddle 2019
Order Solifugae	1	3	300.0%	0	4	43%	Cushing and Brookhart 2019
Subclass Acari	1917^2^	2999	56.6%	?	6629	31%	Beaulieu et al. 2019
**Total Arachnida**	**3220**	**4548**	**41.2**%	**5.7% (89)^3^**	**6982–7032**	**39**%	
**Subphylum Myriapoda**							
Class Chilopoda	30	54	80.0%	31.5% (17)	40	57%	Langor et al. 2019
Class Diplopoda	47	66	40.4%	31.8% (21)	29	70%	Langor et al. 2019
Class Pauropoda	0	23	–	17.4% (4)	17	58%	Langor et al. 2019
Class Symphyla	1	2	100.0%	100.0% (2)	7	22%	Langor et al. 2019
**Total Myriapoda**	**78**	**145**	**85.9**%	**30.3% (44)**	**93**	**61**%	
**Subphylum Hexapoda**							
**Class Entognatha**							
Subclass Collembola	195^4^	470	141.0%	?	180–204	70–72%	Turnbull and Stebaeva 2019; M Turnbull pers. comm.
Order Diplura	2	6	300.0%	?	10–12	33–38%	Sikes 2019
Order Protura	3	9	300.0%	?	10	47%	Sikes 2019
**Total Entognatha**	**200**	**485**	**142.50**%	?	**200–226**	**68–71**%	
**Class Insecta**							
Order Archaeognatha^5^	3	8	167.7%	25.0% (2)	8	50%	Bowser 2019
Order Zygentoma^6^	3^7^	4	33.3%	100.0% (4)	4	50%	Sweetman and Kulash 1944; Appendix I
Order Ephemeroptera	301	335	11.3%	0	66	84%	Jacobus 2019
Order Odonata	194	214	10.3%	0	15	93%	Cannings 2019
Order Plecoptera	250	267	6.8%	0	34	89%	Kondratieff et al. 2019
Order Orthoptera^8^	205^9^	235	14.6%	4.3% (10)	15	94%	Miskelly and Paiero 2019
Order Phasmida^10^	1	1	0	0	1	50%	Miskelly and Paiero 2019
Order Dermaptera	5	6	20.0%	66.7% (4)	0	100%	Miskelly and Paiero 2019
Order Grylloblattodea^11^	2	2	0	50.0% (0)	2	50%	Schoville 2019
Order Blattodea^12^	14	18	28.6%	50.0% (9)	6–8	69–75%	Miskelly and Paiero 2019
Order Mantodea^12^	3	3	0	66.7% (2)	1	75%	Miskelly and Paiero 2019
Order Hemiptera	3079	4011	30.3%	10.1% (405)	589	87%	Foottit et al. 2019
Order Thysanoptera	102	147	44.1%	19.0% (28)	255	37%	Foottit and Maw 2019
‘Psocoptera’^13^	72	108	50.0%	15.7% (17)	67	62%	Anonby 2019
‘Phthiraptera’^14^	362	463	27.9%	8.9% (41)	361^15^	56%	Galloway 2019a
Order Hymenoptera	6028^16^	8757	45.3%	4.6% (402)^17^	10,366–10,391	46%	A Bennett et al. 2019a
Order Coleoptera^18^	6742	8302	23.1%	7.7% (639)	1078–1284	87–89%	Brunke et al. 2019
Order Strepsiptera^18^	6	27	333.3%	0	19	59%	Straka 2019
Order Raphidioptera	7	8	14.3%	0	4	67%	Blades 2019c
Order Neuroptera	75	101	34.7%	6.9% (7)	>48	<68%	Blades 2019b
Order Megaloptera	16	18	12.5%	0	7	72%	Liu 2019
Order Diptera	7056^19^	9620	36.3%	1.5% (147)^17^	5205–20,458	32–65%	Savage et al. 2019
Order Mecoptera	22	25	13.6%	0	>18	<58%	Blades 2019a
Order Siphonaptera	151^20^	154	2.0%	3.9% (6)	23	87%	Galloway 2019b
Order Lepidoptera	4107^21^	5455	32.8%	3.8% (207)	1400	80%	Pohl et al. 2019
Order Trichoptera	546	636	16.7%	0	129–181	78–83%	Sheffield et al. 2019
**Total Insecta**	**29,352**	**38,925**	**32.6**%	**5.0% (1931)**	**19,721–35,259**	**52–66**%	
**Total Terr. Arthropods**	**32,850**	**44,103**	**34.3**%	**5.1% (2064)^22^**	**26,990–42,604**	**51–62**%	

^1^Dondale (1979) reported 1256 species but later discovered an enumeration error; thus the 1979 number should have been 1249 (R Bennett et al. 2019b). ^2^The total for 1979 included both described and undescribed species and it is not possible to now determine the proportion that represented described species. Furthermore, in 1979, 1915 species were reported but this excluded two additional species mentioned in footnotes but not captured in the tally. See Beaulieu et al. (2019) for more details. ^3^Acari were excluded from the calculation of percent of Arachnida that is non-native. ^4^Richards (1979) estimated the number of Collembola species that should occur in Canada at 520. He did not do a tally based on available data. However, Christiansen and Bellinger (1980-81) reported 195 species from Canada and this more closely reflects the known species in 1979 so is used as an approximation of the known diversity at the time. ^5^Archaeognatha was called Microcoryphia in 1979. ^6^Zygentoma was called Thysanura in 1979. ^7^Although only two species of Zygentoma were reported by Tomlin (1979), a third species was known from Canada but the record was missed by Tomlin (see details in Appendix I). ^8^Includes Grylloptera which was recognized as a separate order in 1979. ^9^The total number of Orthoptera + Grylloptera species reported in 1979 was 217; however, this likely included subspecies. The number of species was revised downward to 205 to represent known species at the time (see Miskelly and Paiero 2019). ^10^Phasmida was called Cheleutoptera in 1979. ^11^Grylloblattodea was called Notoptera in 1979. ^12^Blattodea was called Dictyoptera (misspelled Dictuoptera) in 1979, and then included the Mantodea which is now a separate order. ^13^Psocoptera is no longer considered an order but is now recognized as part of the order Psocodea, but it is herein reported on separately. ^14^In 1979, there were two orders of lice recognized, Phthiraptera and Mallophaga. Currently all lice are placed in Phthiraptera which is now considered a part of the order Psocodea, but is herein reported on separately. ^15^Galloway (2019a) did not estimate undescribed species. This number represents described species likely to be in Canada but yet undocumented. ^16^Masner et al. (1979) neglected to include ca. 80 species of Eurytomidae known at the time; however, this omission is roughly balanced by the apparent overestimate of the number of species of Platygastroidea, Ceraphronoidea, Bethylidae, and Pompilidae because undescribed species were included for all of those groups (A Bennett et al. 2019a). Thus, it is assumed that the total number of species actually known at the time was coincidentally close to that reported (6028) despite the enumeration errors. ^17^Counts of non-native species for Diptera and Hymenoptera are based on information extracted from primary literature and in consultation with taxonomists. This information resides in an unpublished database (D Langor unpubl. data). ^18^In 1979, Coleoptera included what is now the order Strepsiptera. ^19^In 1979, 7058 species were reported from Canada but there was an addition error. ^20^Revised downward from 180 species reported by Holland (1979) as his tally included subspecies and taxa from Greenland (see Galloway (2019b) for more details). ^21^Revised downward from 4692 species reported by Munroe (1979) as his estimates included enumeration errors (see Pohl et al. (2019) for details). ^22^Acari and Entognatha were excluded from the calculation of percent of terrestrial arthropods that is non-native.

**Table 2.** Number of Barcode Index Numbers (BINs; Ratnasingham and Hebert 2013) reported for terrestrial arthropods in Canada and the percent of families with assigned BINs. Data were extracted from each of the faunal papers published in Langor and Sheffield (2019) (see Table 1 for references for each taxon). BIN data were originally obtained from the Barcode of Life Data System (www.boldsystems.org).

Taxon	No. families with described species	Percent (no.) of families with BINs	No. BINs available for Canadian species	Ratio of BINs to described species
**Class Arachnida**				
Order Araneae	45	91%	1623	1.10
Order Opiliones	9	89%	64	1.78
Order Pseudoscorpiones	8	75%	46	1.84
Order Scorpiones	1	100%	1	1.00
Order Solifugae	1	100%	1	0.33
Subclass Acari	269	67%	7462	2.49
**Total Arachnida**	**333**	**71**%	**9197**	**2.02**
**Subphylum Myriapoda**				
Class Chilopoda	8	63%	60	1.11
Class Diplopoda	18	72%	65	0.98
Class Pauropoda	2	0%	0	0.00
Class Symphyla	2	100%	4	2.00
**Total Myriapoda**	**30**	**67**%	**129**	**0.89**
**Subphylum Hexapoda**				
**Class Entognatha**				
Subclass Collembola	23	74%	1265	2.69
Order Diplura	2	50%	6	1.00
Order Protura	2	50%	3	0.33
**Total Entognatha**	**27**	**70**%	**1274**	**2.63**
**Class Insecta**				
Order Archaeognatha	2	100%	10	1.25
Order Zygentoma	1	100%	2	0.50
Order Ephemeroptera	21	67%	328	0.98
Order Odonata	10	90%	150	0.71
Order Plecoptera	9	100%	166	0.62
Order Orthoptera	12	75%	157	0.67
Order Phasmida	1	100%	1	1.00
Order Dermaptera	3	100%	4	0.67
Order Grylloblattodea	1	100%	1	0.50
Order Blattodea	5	80%	13	0.72
Order Mantodea	1	100%	2	0.67
Order Hemiptera	86	80%	3275	0.82
Order Thysanoptera	6	67%	338	2.30
‘Psocoptera’	18	100%	162	1.50
‘Phthiraptera’	15	47%	13	0.03
Order Hymenoptera	83	90%	18,454	2.11
Order Coleoptera	120	87%	5750	0.69
Order Strepsiptera	5	80%	3	0.11
Order Raphidioptera	2	100%	10	1.25
Order Neuroptera	10	80%	141	1.40
Order Megaloptera	2	100%	10	0.56
Order Diptera	117	94%	29,583	30.75
Order Mecoptera	4	100%	24	0.96
Order Siphonaptera	7	43%	22	0.14
Order Lepidoptera	81	95%	5842	1.07
Order Trichoptera	25	92%	610	0.96
**Total Insecta**	**647**	**87**%	**65,071**	**1.67**
**Total Terr. Arthropods**	**1037**	**81**%	**75,671**	**1.72**

The association of BINs with known morphological species is ongoing and progress is highly variable from group to group. In most groups there are still many BINs that have not been assigned to species. The percent of described species in the Canadian fauna that currently have associated BINs is also highly variable amongst groups and has not been calculated for many groups. Of the moderately-to-highly diverse groups, at one extreme 92% of the described Araneae (1477 species) have associated BINs (R Bennett et al. 2019b) while at the other extreme only ca. 10% of described Acari (2999 species) have associated BINs (Beaulieu et al. 2019). DNA barcode data have proven to be highly informative to resolve taxonomic issues (e.g., cryptic species, synonymy) and phylogenetic relationships (e.g., Hebert et al. 2004, Hajibabaei et al. 2007); however, as this database expands and is explored in detail (e.g., association of BINs with putative morphological species), its value as a tool to enhance documentation of the Canadian fauna will continue to grow rapidly.

## Distribution

**Figure 1.**
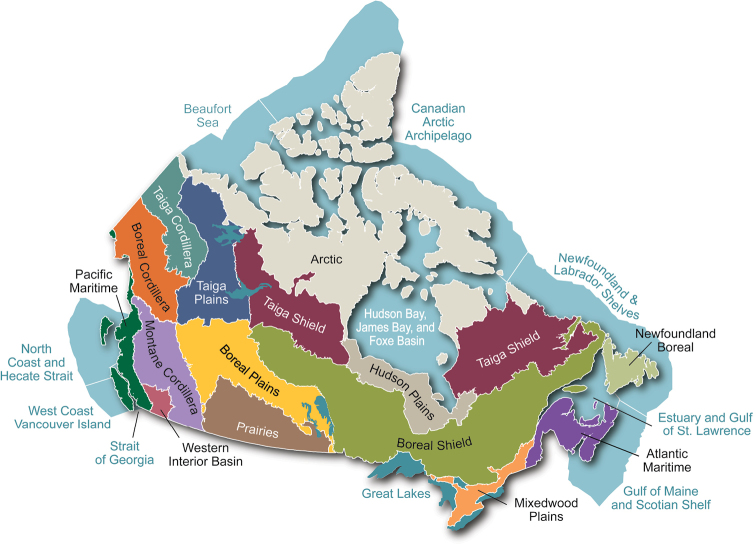
Terrestrial ecozones of Canada as included in the Canadian Biodiversity Ecosystem Status and Trends 2010 report (Federal, Provincial, and Territorial Governments of Canada 2010). [Reprinted with permission from Environment and Climate Change Canada]

Although documentation of the composition of the terrestrial arthropod fauna of Canada is an enormous challenge, understanding the geographic distribution of each species within the country poses an even greater challenge. Many species recorded from Canada are known from only one or a few localities, and this is a reflection of several compounding factors: the large size of the country, much of which is difficult to access (e.g., northern areas, alpine and subalpine habitats); the relatively sparse distribution of historical biological survey activities across the country, with the highest concentration in southern regions; and the relatively small number of people trained to expertly identify collected material resulting in enormous backlogs of unidentified material in practically every terrestrial arthropod collection in the country. Despite the challenges of understanding the distribution of species, Canada and its 13 provincial/territorial jurisdictions are required to report on the conservation status of its native biota every five years, and this requires knowledge about which native species occur in each jurisdiction and how widespread each species is within the jurisdiction (CESCC 2016). However, for the Biota of Canada publication (Langor and Sheffield 2019), where species diversity is reported at the family level, and therefore geopolitical affiliations are not so important, authors were asked to report distribution at the ecozone level as this is more ecologically meaningful. Ecozone designations and boundaries, as depicted in the Ecosystem Status and Trends Report (Federal, Provincial, and Territorial Governments of Canada 2010; Fig. 1), were chosen as the ecological template on which to describe family distributions in the Biota of Canada report. This map reflects the spatial representation of ecozones in Canada the last time that biodiversity in the country was assessed at a national level. In general, the distributions of families are relatively well known compared to those of species; however, even at the family level there are many uncertainties about distribution.

## Conservation

At the time that *Canada and its insect fauna* was written, initiatives focused on species at risk and conservation were in their infancy in Canada. The Committee on the Status of Endangered Wildlife in Canada (COSEWIC) was formed in 1977 but it was not until 2003 that the Species at Risk Act (SARA) was passed and COSEWIC was designated as the national body for identifying and assessing species status. Originally, COSEWIC’s mandate covered only vertebrates and vascular plants, but this expanded in 1994 to cover other groups, including Lepidoptera, and again expanded in 2003 to include other arthropods (Government of Canada 2017). COSEWIC's designations, which are not by themselves legally binding, are taken into consideration by the Government of Canada in establishing the legal list of Species At Risk. As of 2017, 68 terrestrial arthropod species and subspecies (67 insects and one spider) have COSEWIC status in Canada (Table 3): four are extirpated, 42 are endangered, eight are threatened, and 15 are of special concern (one species is listed as endangered in one jurisdiction and of special concern in another). Half of the taxa are Lepidoptera (34) and the remainder are Coleoptera (10), Hymenoptera (9), Odonata (8), Orthoptera (3), Diptera (2), Hemiptera (1), and Araneae (1). Fifty species or subspecies have been designated under SARA as ‘Species At Risk’, including one species that was designated ‘not at risk’ by COSEWIC (Table 3).

The Canadian Endangered Species Conservation Council’s National General Status Working Group (NGSWG), which has representation from all provincial and territorial governments in Canada as well as the federal government, plays a major role in evaluating and prioritizing species to recommend to COSEWIC for consideration (although recommendations may also come from other sources). The NGSWG engages experts in Canada to consider all available scientific evidence and use an objective process developed by NatureServe (www.natureserve.org) to assess the conservation status of species within each province and territory and in Canada as a whole. Every five years starting in 2000, the NGSWG has assessed the conservation status of many species for each province and territory and published its Wild Species report. In the most recent report, *Wild Species 2015* (CESCC 2016), the following terrestrial arthropod groups were assessed: Araneae, Ephemeroptera, Odonata, Plecoptera, Orthoptera, Neuroptera, Coleoptera, Lepidoptera, Trichoptera, some Hymenoptera, Mecoptera, and some Diptera (Table 4). In that report, 18,692 terrestrial arthropod species and subspecies were assessed, 17,734 of which were native taxa. Of the native taxa (species and subspecies), one was presumed ‘extirpated’, 37 (0.2%) were considered ‘possibly extirpated’, 177 (1.0%) were designated ‘critically imperiled’, and 261 (1.5%) were designated ‘imperiled’. Of these 476 taxa, 53 were categorized as ‘most at risk’ and these represent taxa that have high priority for consideration by COSEWIC. Almost 50% of native taxa assessed had insufficient data to allow conservation ranks to be assigned. Among these unranked taxa there are likely many additional species at high risk, especially those that could have highly specialized habitats and very limited distributions, but their poor representation in collections limits ability to assess them according to current methods.

**Table 3.** Species and subspecies of terrestrial arthropods designated by the Committee on the Status of Endangered Wildlife in Canada (COSEWIC) and those listed as ‘at risk’ under the Species At Risk Act (SARA). Data were extracted from the database maintained by Government of Canada (2018).

Species	Higher classification	COSEWIC status	Designation status by SARA	Historical Range of occurrence^1^
*Nicrophorusamericanus* (Olivier)	Insecta: Coleoptera: Silphidae	extirpated	extirpated	ON, QC
*Callophrysirus* (Godart)	Insecta: Lepidoptera: Lycaenidae	extirpated	extirpated	ON
*Lycaeidesmelissasamuelis* (Nabokov)	Insecta: Lepidoptera: Lycaenidae	extirpated	extirpated	ON
*Euchloeausonidesinsulanus* (Guppy & Shepard)	Insecta: Lepidoptera: Pieridae	extirpated	extirpated	BC
*Cicindelamarginipennis* Dejean	Insecta: Coleoptera: Carabidae	endangered	endangered	NB
*Cicindelaparowanawallisi* Calder	Insecta: Coleoptera: Carabidae	endangered	endangered	BC
*Cicindelapatruela* Dejean	Insecta: Coleoptera: Carabidae	endangered	endangered	ON, QC
*Coccinellanovemnotata* Herbst	Insecta: Coleoptera: Coccinellidae	endangered	no status	AB, BC, MB, ON, QC, SK
*Sanfilippodytesbertae* Roughley & Larson	Insecta: Coleoptera: Dytiscidae	endangered	endangered	AB
*Brychiushungerfordi* Spangler	Insecta: Coleoptera: Haliplidae	endangered	endangered	ON
*Efferiaokanagana* Cannings	Insecta: Diptera: Asilidae	endangered	endangered	BC
*Bombusaffinis* Cresson	Insecta: Hymenoptera: Apidae	endangered	endangered	ON, QC
*Bombusbohemicus* (Seidl)	Insecta: Hymenoptera: Apidae	endangered	endangered	all but NU
*Epeoloidespilosulus* (Cresson)	Insecta: Hymenoptera: Apidae	endangered	endangered	NS
*Erynnismartialis* (Scudder)	Insecta: Lepidoptera: Hesperiidae	endangered	no status	MB, ON, QC
*Erynnispersiuspersius* (Scudder)	Insecta: Lepidoptera: Hesperiidae	endangered	endangered	ON
*Hesperiacoloradooregonia* (Edwards)	Insecta: Lepidoptera: Hesperiidae	endangered	no status	BC
*Hesperiadacotae* (L.)	Insecta: Lepidoptera: Hesperiidae	endangered	endangered	MB, SK
*Hesperiaottoe* (Edwards)	Insecta: Lepidoptera: Hesperiidae	endangered	endangered	MB
*Oarismapoweshiek* (Parker)	Insecta: Lepidoptera: Hesperiidae	endangered	threatened	MB
*Plebejussaepiolusinsulanus* Blackmore	Insecta: Lepidoptera: Lycaenidae	endangered	endangered	BC
*Satyriumbehrii* (Edwards)	Insecta: Lepidoptera: Lycaenidae	endangered	endangered	BC
*Satyriumsemiluna* Klots	Insecta: Lepidoptera: Lycaenidae	endangered	endangered	AB, BC
*Anartaedwardsii* (Smith)	Insecta: Lepidoptera: Noctuidae	endangered	no status	BC
*Copablepharonfuscum* Troubridge & Crabo	Insecta: Lepidoptera: Noctuidae	endangered	endangered	BC
*Copablepharonlongipenne* Grote	Insecta: Lepidoptera: Noctuidae	endangered	endangered	AB, MB, SK
*Papaipemaaweme* (Lyman)	Insecta: Lepidoptera: Noctuidae	endangered	endangered	ON
*Pyrrhiaaurantiago* (Guenée)	Insecta: Lepidoptera: Noctuidae	endangered	no status	ON
*Schiniaavemensis* Dyar	Insecta: Lepidoptera: Noctuidae	endangered	endangered	AB, MB, SK
*Schiniabimatris* Harvey	Insecta: Lepidoptera: Noctuidae	endangered	endangered	MB
*Coenonymphanipisiquit* McDunnough	Insecta: Lepidoptera: Nymphalidae	endangered	endangered	NB, QC
*Danausplexippus* (L.)	Insecta: Lepidoptera: Nymphalidae	endangered	special concern	all but NT, YT
*Euphydryasedithataylori* (Edwards)	Insecta: Lepidoptera: Nymphalidae	endangered	endangered	BC
*Praysatomocella* (Dyar)	Insecta: Lepidoptera: Plutellidae	endangered	no status	ON
*Prodoxusquinquepunctellus* (Chambers)	Insecta: Lepidoptera: Prodoxidae	endangered	no status	AB
*Tegeticulacorruptrix* Pellmyr	Insecta: Lepidoptera: Prodoxidae	endangered	endangered	AB
*Tegeticulayuccasella* (Riley)	Insecta: Lepidoptera: Prodoxidae	endangered	endangered	AB
*Apodemiamormo* (Felder & Felder)^2^	Insecta: Lepidoptera: Riodinidae	endangered	endangered	BC
*Hemileucanuttallii* (Strecker)	Insecta: Lepidoptera: Saturniidae	endangered	no status	BC
*Hemileuca* sp.	Insecta: Lepidoptera: Saturniidae	endangered	endangered	ON
*Somatochlorahineana* Williamson	Insecta: Odonata: Corduliidae	endangered	endangered	ON
*Gomphusventricosus* (Walsh)	Insecta: Odonata: Gomphidae	endangered	endangered	NB
*Phanogomphusquadricolor* (Walsh)	Insecta: Odonata: Gomphidae	endangered	endangered	ON
*Stylurusamnicola* (Walsh)	Insecta: Odonata: Gomphidae	endangered	endangered	ON
*Styluruslaurae* Williamson	Insecta: Odonata: Gomphidae	endangered	no status	ON
*Stylurusolivaceus* (Selys)	Insecta: Odonata: Gomphidae	endangered	endangered	BC
*Cicindelaformosagibsoni* Brown	Insecta: Coleoptera: Carabidae	threatened	threatened	AB, SK
*Omusaudouini* Reiche	Insecta: Coleoptera: Carabidae	threatened	threatened	BC
*Bombusoccidentalisoccidentalis* (Greene)	Insecta: Hymenoptera: Apidae	threatened	no status	AB, BC, SK
*Lasioglossumsablense* Gibbs	Insecta: Hymenoptera: Halictidae	threatened	threatened	NS
*Grammiacomplicata* Walker	Insecta: Lepidoptera: Erebidae	threatened	no status	BC
*Euphyesvestrisvestris* (Boisduval)	Insecta: Lepidoptera: Hesperiidae	threatened	threatened	BC
*Schiniaverna* Hardwick	Insecta: Lepidoptera: Noctuidae	threatened	threatened	AB, MB, SK
*Trimerotropishuroniana* Walker	Insecta: Orthoptera: Acrdidiae	threatened	no status	ON
*Gnaphosasnokomish* Platnick & Shadab	Arachnida: Araneae: Gnaphosidae	special concern	special concern	BC
*Coccinellatransversoguttata* Faldermann	Insecta: Coleoptera: Coccinellidae	special concern	no status	all jurisdictions
*Germariaangustata* (Zetterstedt)	Insecta: Diptera: Tachinidae	special concern	special concern	YT
*Aflexiarubranura* (DeLong)	Insecta: Hemiptera: Cicadellidae	special concern	no status	MB, ON
*Bombusoccidentalismckayi* Ashmead	Insecta: Hymenoptera: Apidae	special concern	no status	BC, NT, YT
*Bombuspensylvanicus* (De Geer)	Insecta: Hymenoptera: Apidae	special concern	no status	ON, QC
*Bombusterricola* Kirby	Insecta: Hymenoptera: Apidae	special concern	special concern	all but NU
*Dielispilipes* (Saussure)	Insecta: Hymenoptera: Scoliidae	special concern	no status	BC
*Copablepharongrandis* (Strecker)	Insecta: Lepidoptera: Noctuidae	special concern	special concern	AB, MB, SK
*Limenitisweidemeyerii* Edwards	Insecta: Lepidoptera: Nymphalidae	special concern	special concern	AB
*Apodemiamormo* (Felder & Felder)^2^	Insecta: Lepidoptera: Riodinidae	special concern	special concern	SK
*Argiavivida* Hagen	Insecta: Odonata: Coenagrionidae	special concern	no status	AB, BC
*Ophiogomphushowei* Bromley	Insecta: Odonata: Gomphidae	special concern	special concern	NB, ON
*Hypochloraalba* Dodge	Insecta: Orthoptera: Acrdidiae	special concern	special concern	AB, MB, SK
*Melanoplusmadeleineae* Vickery & Kevan	Insecta: Orthoptera: Acrdidiae	special concern	no status	QC
*Politessonora* (Scudder)	Insecta: Lepidoptera: Hesperiidae	not at risk	special concern	BC

^1^ Jurisdictional abbreviations are as follows: AB – Alberta; BC – British Columbia; NB – New Brunswick; NS – Nova Scotia; NT – Northwest Territories; NU – Nunavut; MB – Manitoba; ON – Ontario; QC – Quebec; SK – Saskatchewan; YT – Yukon. ^2^*Apodemiamormo* (Felder & Felder) is listed twice because of different species designation in BC and SK.

**Table 4.** Number of species of terrestrial arthropods and their conservation status as assessed by the Canadian Endangered Species Conservation Council’s National General Status Working Group in its Wild Species 2015 report (CESCC 2016).

Taxon	No. species assessed	No. native species	Conservation status				No. high priority species	No. with insufficient data
			Extirpated	Possibly extirpated	Critically imperiled	Imperiled		
Araneae	1399	1328	0	0	7	37	4	460
Ephemeroptera	342	342	0	0	1	2	1	266
Odonata	213	212	0	1	11	15	0	4
Plecoptera	293	293	0	0	0	0	0	193
Orthoptera	269	255	0	8	12	12	6	29
Neuroptera	101	95	0	0	0	2	0	73
Coleoptera	7963	7339	0	22	78	53	19	3624
Hymenoptera: Formicidae	212	197	0	2	0	0	0	53
Hymenoptera: Anthophila	805	787	0	0	4	30	3	349
Hymenoptera: Vespidae	101	95	0	0	12	19	0	6
Trichoptera	688	688	0	0	0	1	0	470
Lepidoptera	5257	5066	1	2	33	56	15	3015
Mecoptera	25	25	0	0	1	2	1	8
Diptera: Simuliidae	160	160	0	0	1	4	1	42
Diptera: Culicidae	80	77	0	0	0	0	0	12
Diptera: Tabanidae	144	144	0	1	4	7	0	22
Diptera: Bombyliidae	116	116	0	1	9	6	0	48
Diptera: Syrphidae	524	515	0	0	4	15	3	189
**Total**	**18,692**	**17,734**	**1**	**37**	**177**	**261**	**53**	**8863**

## How many species are in Canada?

It is a common phenomenon that people (usually taxonomists) who make an effort to estimate the number of unknown species within an area tend to be conservative, especially for groups that have large numbers of undocumented species (Danks 1979a). Certainly the vast majority of estimates provided in Danks (1979a) proved to be conservative 40 years later. Thus, many (perhaps most) estimates of undocumented species provided in the Biota of Canada papers (summarized in Table 1) are also conservative, and some authors state this explicitly. Some estimates are given as rounded numbers, some as ranges, some are open-ended (e.g., >48 Neuroptera), but most are given as a specific number. In the latter case, these numbers should not be interpreted as precise but rather as ‘reasoned approximations’. For each terrestrial arthropod group, the authors described how the estimates were made, so individual papers should be consulted to understand the estimation processes. In at least two cases (Coleoptera, Diptera), authors consulted broadly among experts to gather a wide variety of opinions. In general, authors considered three main kinds of information/data in formulating estimates: literature records, undescribed material known to them, and BINs. Firstly, based on the published literature (including on-line databases/catalogues) it is evident that there are many species that occur in adjacent parts of the USA but are not yet known from Canada, even though the appropriate habitats/hosts occur here and the climate is suitable. It is anticipated that a large proportion of these species will eventually be discovered in Canada. Furthermore, non-native species that are established in the USA and are evidently spreading towards Canada, and can tolerate the Canadian climate, were also considered. Secondly, for most groups, authors also considered Canadian specimens that they had seen (or were aware of) in collections that likely represent undescribed species. Of course, without taxonomic revisions the number of undescribed species with populations in Canada can only be roughly estimated. Thirdly, given the relatively high concordance between BINs and species for most groups of terrestrial arthropods (see discussion above), authors gave consideration to the discrepancy between number of BINs and number of described species in families where BINs outnumbered known species. While generally every BIN does not represent a unique species, nor does every species have a unique BIN, the degree of concordance between BINs and species within taxon groups (e.g., families) can be used to approximate undocumented species diversity. None of these estimation methods represent an exact science, but together they lend credence to estimates and are therefore of more value than an under-considered guess. Furthermore, the estimates were provided by those who best know the Canadian fauna.

Altogether, an estimated ca. 27,000 to 42,600 additional undocumented terrestrial arthropod species are expected to occur in Canada, meaning that the country is home to between ca. 71,100 and 86,700 species. This is 9–32% higher than the species diversity estimated in 1979 (65,507 species; see Table 1 for adjusted described species totals for 1979 and see Danks (1979a, b) for estimates of undocumented species). Likely, 40 years from now, the experts of the time will see that our most current projections were also underestimates. Between 38% and 49% of the expected Canadian fauna remains undocumented. In 1979, an estimated 50% of the terrestrial arthropod fauna was unknown. Some may be inclined to point out that we may not be much better off now than we were in 1979 in terms of the percentage of our fauna that is documented. However, the fact is that more than 11,200 additional species have been documented from Canada during the last 40 years, a very significant achievement! Furthermore, there has been great advancement in understanding the true diversity of species in our country which has led to the realization that Canada is far more biodiverse than anticipated 40 years ago. The challenge is that there is plenty of work left to do.

Comparison of known (described) species richness to estimated species richness for each terrestrial arthropod group is helpful to understand the relative degree to which taxa are known (Table 1). Among the seven most diverse groups in Canada (groups with >1000 described species), the Acari is most poorly known with less than one third of species described. The percent of described Diptera ranges from 32% to 65%, so in the worse-case scenario flies are about as poorly known as mites; however, unlike the mites the largest proportion (perhaps even the majority) of undocumented flies are in one family [Cecidomyiidae, with 1000 to 16,000 additional species expected (Savage et al. 2019)]. Less than half (ca. 46%) of Hymenoptera in Canada are described. Of the moderately diverse groups (50–1000 species expected in Canada), Thysanoptera (37%) and Phthiraptera (56%) are relatively poorly known. In the case of Phthiraptera the proportion of the fauna that is known as much less than 56% as undescribed species were not estimated by Galloway (2019a) because there was no reasonable way to do so. Several groups with low diversity in Canada (< 50 species expected) are poorly known and most of these represent soil- and litter-dwelling species, including Symphyla (22%), Diplura (33–38%), Solifugae (43%), Protura (47%), Pseudoscorpiones (48%), and Archaeognatha (50%). The best known groups are the Scorpiones (100%, 1 species) and Dermaptera (100%, 6 species), but both have very low diversity and are well surveyed in Canada because they are conspicuous. Among the moderately-to-highly diverse groups, the best known are Orthoptera (94%) and Odonata (93%) which contain mainly large and conspicuous species. Other well-known groups are Plecoptera (89%), Coleoptera (87–89%), Hemiptera (87%), and Siphonaptera (87%). In particular, aquatic groups seem best known and soil- and litter-dwelling groups least known.

## Looking to the future

With several tens of thousands of terrestrial arthropod species remaining to be discovered in Canada (many of them requiring description), and the distribution and conservation status of most of the currently documented species poorly known, we cannot rest on the laurels of our collective endeavour over the last four decades. There is much to do before our knowledge about diversity and distribution of the Canadian terrestrial arthropod fauna is as good as that which currently exists for the fauna of western Europe, likely the best documented large-scale regional fauna in the world and representing a state-of-knowledge that is reasonable to aspire towards. There are several key activities that Canada needs to continue investing in to ensure that work on documenting the terrestrial arthropod biota of Canada continues at a pace at least equivalent to that of the last 40 years, and hopefully at a much faster pace given mounting pressures on the environment and its constituent species and ecological communities. These activities are not specific to terrestrial arthropods but are broadly relevant to most groups of biota in Canada. To comprehensively document biodiversity in Canada it is necessary to survey it well throughout the country, continue to build the taxonomic/phylogenetic foundation to define and identify species and their relationships, and manage the wealth of data and information to allow ready access and use, and each of these activities is herein briefly discussed to summarize needs and provide some suggestions. Of course, these activities require financial resources and expertise so biodiversity science stakeholders in Canada must continue to work to ensure that the values and outcomes of these activities are appreciated by society in general and are clearly linked to government priorities and policies to ensure that their relevance is indisputable and that the rationale for investment is irrefutable. This is not a trivial job and will only be sufficiently successful through strategic coordination across the community of stakeholders, and there is much room for improving stakeholder engagement and strategic planning.

### Surveys

**Figure 2.**
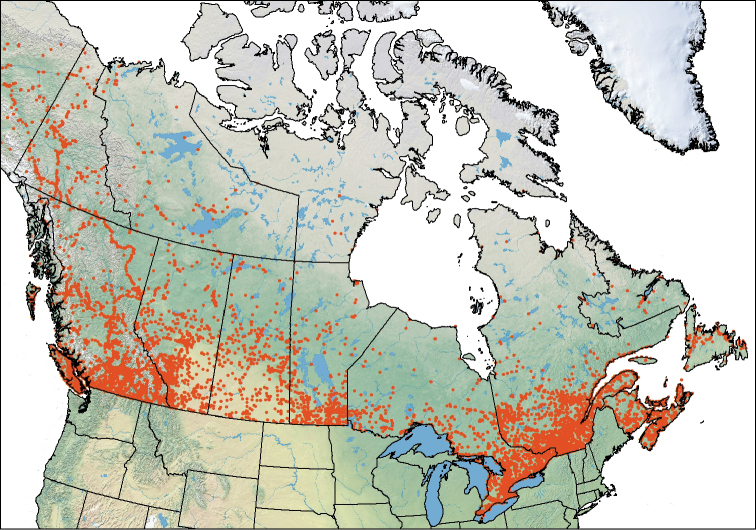
Collection points for the 375 species of Cerambycidae (Coleoptera) in Canada based on 81,555 records extracted from 106 Canadian and USA collections (Bousquet et al. 2017).

The immense physical size of Canada and the difficult and expensive access to large portions of the country (e.g., high latitudes and high altitudes) means that the vast majority of survey effort has been done in the south of the country and around major population centers and along major roads further north. This survey bias is exemplified by a map (Figure 2) of 81,555 collection points for the 375 species of Cerambycidae (Coleoptera) in Canada that were extracted from 106 Canadian and USA collections (see list in Bousquet et al. 2017). Even in southern parts of the country, there are habitats that are under-sampled for terrestrial arthropods. Thus, whenever there is concerted survey activity, the results in terms of new jurisdictional records can be astounding. For example, even following many years of sampling of Coleoptera in the Maritime Provinces, over 300 new beetle records were recently reported for New Brunswick (Webster et al. 2016). Similarly, sampling in Newfoundland and Labrador, mainly from 2008 to 2014, resulted in 119 new provincial records, six new Canadian records, and 34 new species of aleocharine rove beetles (Klimaszewski et al. 2011, 2016) and at least 90 new provincial records for other subfamilies of rove beetles (D Langor unpubl. data). As another example, a long-term survey effort in Waterton Lakes National Park since 2005 has resulted in many new Canadian and provincial records and new species of insects (Pohl et al. 2018; G Pohl, J Klimaszewski, and D Langor unpubl. data). Surveys of spiders in British Columbia, including at high elevations, in recent years have resulted in many new Canadian and provincial records (R Bennett et al. 2019b). There are many other examples of ongoing short- and long-term surveys in Canada that continue to yield new records of species. However, there are limited resources for surveying and the country is large, so prioritization and increased efficiency of survey effort is needed. Consideration should be given to where survey resources are best invested. Focus on biodiversity hotspots and threatened habitats (e.g., remnants of Carolinian forests and native grasslands), regions where there has been low sampling effort to date (e.g., alpine and subalpine zones, Arctic and Taiga ecozones), and undersampled habitats (e.g., hot springs, soils, saproxylic habitats, bird and mammal nests, and the bodies of vertebrates and invertebrates which are inhabited by many species, especially mites) may yield more value per unit of effort than further investment in surveys in areas and habitats that are relatively well sampled. There ought to be more discussion within the biodiversity community in Canada to prioritize and organize sampling efforts to make most efficient use of limited resources. This may also give direction and encouragement to growing numbers of citizen scientists who have the potential to immensely enhance biological surveys (see below).

In recent years, various survey initiatives have been developed to enhance sampling of biodiversity, including terrestrial arthropods. Since 2008, the Centre for Biodiversity Genomics (University of Guelph) has used its BIObus and teams to visit many biodiversity hotspots in Canada to sample specimens for DNA barcoding, and this effort has yielded hundreds of thousands of specimens and ca. 20,000 species (https://biobus.ca). Each year since 2007, the Alberta Biodiversity Monitoring Institute (ABMI) has systematically surveyed soil fauna across the province on a 20 km × 20 km plot grid, resulting in 400 soil samples each year from which invertebrates were extracted for monitoring purposes (T Cobb pers. comm.). This work has resulted in an enormous amount of information particularly about oribatid mite diversity and distribution (Walter et al. 2014). Furthermore, Bioblitzes have been organized by organizations such as the Biological Survey of Canada (eleven Bioblitzes since 2001; https://biologicalsurvey.ca) and Bioblitz Canada (35 Bioblitzes across the country in 2017 to mark the Canada 150 celebrations; www.bioblitzcanada.ca). Bioblitzes serve to bring together biodiversity experts and members of the public to focus on sampling the biota in a small region during a short period (normally 2–5 days). These have served to enhance surveys of the country’s biota, bring attention to the importance of understanding Canada’s biodiversity, and foster collaborations between professional biologists, students, and citizen scientists.

Another source of valuable specimens is from trap-based sampling programs established for a specific research or monitoring purpose. Frequently, only a subset of the taxa collected in experiments or for monitoring is utilized and the remainder (often called bycatch or residual) is discarded. Field sampling programs are expensive and often logistically challenging. Therefore, discarded specimens of non-target taxa represent missed opportunities to maximize return on investment, especially when such material is from regions and habitats that are generally poorly sampled. Clearly, saving bycatch has a cost in terms of additional processing time and storage, and when budgets are lean this additional cost can be prohibitive. However, there are also many people who are willing to make an effort to save some bycatch if they know that there is interest in the material by those who will make some effort to prepare and identify it and use the data. Where resource challenges could limit capacity to extract and store bycatch, creative solutions could be found through partnerships between those generating bycatch and those who have interest in it, e.g., through provision of funds for additional costs or in-kind supply of labour, to offset additional processing/storage costs. Opportunities to match supply and demand of bycatch require a communication network that serves as a clearinghouse service that connects people. An organization willing to perform this service could provide added benefit to survey activities in Canada.

Although historically the collection and examination of biological specimens has been the main source of data on presence and distribution of species, and remains the dominant source, increasingly photographs are yielding valuable information about the identity and location of species that on occasion reveals new jurisdiction records. Some of the best known initiatives that crowdsource data from primarily photos are iNaturalist (https://www.inaturalist.org/), which is global in coverage, includes plants, animals and fungi, and has nearly 90,000 users, and BugGuide (https://bugguide.net/node/view/15740) which is North American in scope and focuses on insects and other terrestrial arthropods. There are also initiatives that focus on particular taxa such as moths, e.g., Moth Photographers Group (http://mothphotographersgroup.msstate.edu/) and Mothing and Moth Watching (https://www.facebook.com/groups/137219092972521/). Within Canada there are several similar initiatives that are social-media-based and have much (or almost all) content focused on terrestrial arthropods, e.g., Alberta Bugs and Insects (https://www.facebook.com/groups/782992888444902/), Insects of Newfoundland (https://www.facebook.com/groups/717236451733098/), and NWT Species (https://www.facebook.com/groups/NWTSpecies/). As well, there are email listserves that have a similar purpose, e.g. Albertabugs and Albertaleps, both accessed through the University of Alberta. These initiatives serve two main purposes. First, they promote citizen science by encouraging public curiosity and information sharing and providing them with tangible rewards in terms of feedback from specialists concerning, e.g., identification and biological information. Secondly, on occasion photos reveal new or interesting records or natural history observations. However, identification depends on the quality of the photos and whether the species in question is identifiable based on a habitus, so only a small proportion of photos allow an accurate species determination. Thus, crowdsourcing of data through photographs will continue to provide a relatively small, albeit valuable, contribution to the future documentation of the Canadian fauna. However, through such citizen science initiatives that connect the enthusiastic public with appreciative and encouraging specialists, opportunities are created to encourage and train some ‘citizens’ to become more involved in surveys through the more traditional and data-rich method of collecting and preserving specimens to submit to specialists for identification. There are now cases where citizens who started as ad hoc sources of insect photos are now regularly collecting specimens that are contributing valuable records (G Pohl pers. comm.). The challenge is to encourage more specimen sampling by non-specialists by increasingly connecting specialists with the willing and capable public in mutually rewarding ways. While there is an investment required from the specialist to engage in training, provide some supplies (at least initially), respond to enquiries, provide identifications, etc., the potential for high return on the investment is excellent. More generally, the participation of enthusiastic specialists in public events such as Bioblitzes, science fairs, public lectures, natural history societies, school presentations, etc., and by creating products that have appeal to the ‘nature-curious’ public (e.g., field guides, websites, and videos), can potentially increase the participation of the public in natural science activities, including surveying of the biota.

For more than 41 years, the Biological Survey of Canada (BSC) has played important roles in promoting and fostering survey activities for terrestrial arthropods in Canada and synthesizing and distributing biodiversity information. The strength of the BSC is that it does not have institutional or departmental affiliation and therefore is not directed by top-down pressures to adhere to institutional or political agendas. As a network of frontline biodiversity workers, the collective expertise of the BSC self-organizes to focus on activities that fill important gaps in knowledge on Canada’s biodiversity. The BSC has rallied resources to focus on specific regions (e.g., Yukon, Haida Gwaii, the Arctic, and Newfoundland and Labrador), habitats (e.g., springs, ectoparasites of vertebrates), biotic communities (e.g., grasslands), and topics (e.g., non-native species) that have helped foster focused survey activities, resulted in significant products (e.g., books, scientific papers, newsletters), and greatly improved the state of knowledge of Canada’s terrestrial arthropod biodiversity (see Danks (2017) for the details of some of the accomplishments of the BSC). Particularly remarkable is the fact that in 1979 the BSC successfully engaged the Canadian biodiversity community to review the state of knowledge for terrestrial arthropod diversity in Canada, that resulted in the monograph *Canada and its insect fauna* (Danks 1979b), and is again doing so, 40 years later, through the current Biota of Canada initiative. There remain important roles for the BSC to play in promoting and coordinating national efforts to document the country’s biota in partnership with other biodiversity stakeholders. As it has no institutional constraints or political agenda, the BSC is well-placed to serve as a needs-driven, impartial broker and catalyst to continue to provide focus and foster activities on important knowledge gaps concerning the biota of Canada.

### Taxonomy, diagnostics, and DNA barcodes

It is relatively easy, in terms of time and skills, to sample huge numbers of terrestrial arthropod specimens, especially using traps; however, it can be very time-intensive to identify them, even for taxa for which modern identification tools exist. DNA barcoding is increasingly helping with the identification process if the specimens are of sufficient quality to barcode and when there are comprehensive barcode libraries. However, DNA barcoding (or molecular approaches in general) is not a replacement for traditional morphology-based taxonomy but rather they are complimentary (Roe et al. 2017). Taxonomy has suffered steep declines in recent years in Canada, especially in universities (Packer et al. 2009, Archambault et al. 2010), which is also the global trend (Kim and Byrne 2006). It is therefore necessary to continue Canadian investment in taxonomy that integrates molecular, morphological, and ecological evidence to distinguish species, organize them within a phylogenetic structure, and develop identification tools (e.g., keys, molecular profiles). Without this, we will never be able to document, understand and protect the enormous biodiversity that surrounds us and on which we depend for environmental services and a sound economy.

Survey activities have resulted in accumulation of specimens in collections at a faster rate than they can be processed and identified to species, especially for groups where there are no Canadian specialists or modern identification tools. Canadian and foreign collections contain huge numbers of Canadian specimens that are not prepared or are identified only to genus or higher levels because there are insufficient people to do authoritative identifications and a lack of modern revisions and identification tools. Undoubtedly, a large proportion of the conservatively estimated 27,000 to 42,600 undocumented terrestrial arthropod species in Canada are already represented by specimens that have been collected and now reside in collections, either in containers of preserved, unprepared material or as prepared and labelled specimens. Furthermore, large numbers of valuable records of documented species, even in groups that are well known and have Canadian specialists and modern identification tools, have not yet been recognized because of the huge backlog in diagnostics. The Canadian taxonomic and diagnostic capacity is simply overwhelmed, and this is especially evident for highly diverse and relatively poorly known groups such as Acari, Diptera and Hymenoptera.

During the last 40 years, between 11,000 and 12,000 terrestrial arthropod species were newly documented in Canada (Table 1). With equivalent effort per unit of time in the future, and given that 27,000 to 42,600 additional species (even the upper limit is likely a conservative estimate) are waiting to be discovered, it will take 90–150 years for the fauna to be documented. Although molecular approaches will increase the rate of documentation of the fauna, it will still be decades before we know the identity of species in the country, let alone know their full distribution, habitat associations, etc. This is sobering, especially against a backdrop of rapid ongoing environmental change that is altering habitats and likely species viability in the country. Do we need to document all taxa and assign species names? Are measures of genetic diversity (e.g., BINs) sufficient for some hyper-diverse groups where there are few specialists and no impetus to study them because they are not sufficiently attractive or have little or no adverse effects on humans (e.g., cecidomyiid flies which have 1000–16,000 undocumented species in Canada)? What are the priorities for taxonomic investment? Should priorities be based mainly (or solely) on importance to agriculture, forestry and health as they are today? Can we better harness the potential of citizen scientists to engage in taxonomic/diagnostic activity? More fundamentally, is the value of documenting and understanding diversity in the natural world, the challenges, and trade-offs sufficiently understood by society, funding agencies, and policy-makers to allow appropriate prioritization and sound investment decisions? All of these and other questions require discussion across the full range of biodiversity stakeholders as we collectively try to find the most efficient way forward to document our Canadian biodiversity.

DNA barcoding has made significant contributions to biosystematics and the documentation of the Canadian biota, and its influence will grow as the DNA barcode reference library grows and more people use it to help reconcile taxonomic problems, improve diagnostic capacity and speed, and understand phylogenetic relationships. Already for terrestrial arthropods there are more than 75,000 BINs based on Canadian specimens, but there remains a large job of reconciling BINs with morphological concepts to understand the degree to which DNA barcodes reflect species and to build comprehensive voucher libraries. Improvement of protocols (e.g., better primers) that increase the success rate of barcoding attempts for certain groups (e.g., Brunke et al. 2019) will provide a better return on barcoding investment. Approaches that allow improved DNA recovery and amplification for older specimens and those collected using suboptimal techniques/preservatives will enhance data accumulation. Currently barcoding efforts have not been evenly distributed across the country and sampling equitability is needed to characterize the Canadian biota and its distribution. Especially promising is the rapid development of next generation sequencing approaches that readily allows sampling of many genes (in organelles and the nucleus) through ‘massively parallel sequencing’, and which will increase the utility of molecular data in defining species concepts and relationships and providing diagnostic tools (Roe et al. 2017).

### Species checklists

The development of species checklists is but one facet of the broader realm of specimen and data management wherein there are other important considerations and needs concerning, e.g., biological collections, data standards, and data mobilization; however, these topics have been well covered elsewhere (e.g., Suarez and Tsutsui 2004, Johnson 2017). The values of species checklists and the particular needs of this activity, however, have not been sufficiently discussed and promoted.

Even though checklists are not included in this Biota of Canada Special Issue, most authors relied upon existing checklists or created their own as a basis for summarizing and analyzing species richness data. It is widely appreciated that species checklists, whether hard copy or electronic in nature, represent a useful means of synthesizing and sharing information about diversity and distribution of species. Since 1979, almost all of the most species-diverse terrestrial arthropod groups have been the focus of cataloguing efforts in Canada that have resulted in national checklists that show jurisdictional distributions and provide current nomenclature and classification, e.g., Maw et al. (2000) for Hemiptera (currently being updated; R Foottit pers. comm.), Paquin et al. (2010) for Araneae (with subsequent updates; see R Bennett et al. (2019a) for details), Bousquet et al. (2013) for Coleoptera, Pohl et al. (2018) for Lepidoptera, and A Bennett et al. (in prep.) for Hymenoptera. As well, in recent years, the National General Status Working Group has fostered the development of species lists for several orders of insects, including Ephemeroptera, Odonata, Plecoptera, Orthoptera, Neuroptera, Trichoptera, and Mecoptera as a necessary first step in assessing conservation status of individual species (CESCC 2016). Notably, Acari and Diptera, two of the six largest groups in the country, do not have national or provincial/territorial checklists. The state of knowledge of each these two highly diverse groups is too fragmented and preliminary to yet contemplate production of a national checklist for the entire group, although some portions (e.g., families) of these groups have been catalogued (Beaulieu et al. 2019, Savage et al. 2019).

Checklists of species, whether for a genus, family, order, or class, and whether national in scope or focused on a smaller geographic scale (e.g., province/territory, region, island, ecozone) or habitat, have high value. Checklist development requires synthesis of the body of evidence concerning diversity, classification and nomenclature, and therefore it represents a state-of-knowledge product. As a composite of collective knowledge, the process of creating a checklist tends to rally available expertise to collaborate and consider all available data and information. Checklists also fill federal, provincial and territorial needs as they are required by the NGSWG as the foundational first step in assessing conservation status of species, which is a national obligation. Furthermore, checklists serve to highlight gaps in the state of knowledge that can help prioritize future sampling and taxonomic endeavours. Finally, checklists provide a framework on which to organize new data (e.g., new records, new species, and changes in nomenclature and classification). Having a checklist that is publically available tends to challenge the biodiversity community (both professionals and citizen scientists) to improve on it, and this challenge usually engenders new sampling activity, makes it easy to determine if records are new, and encourages those with new records to make them known.

Checklists are outdated soon after they are published in terms of the included species, jurisdictional distribution, nomenclature, classification, etc. Soon after obtaining a newly published checklist, the knowledgeable user is soon filling the margins with notes concerning new and corrected information, and these notes summed across the community of users represent valuable improvements to the checklist. However, all notes and improvements are not usually available to all other users and thus the improvements of the checklist are not universally available until far into the future (usually decades) when the next edition of the checklist is published. Thus, to keep checklists current they need to be on-line and dynamic so that as new records (or other changes) are discovered, they are quickly vetted within the community of experts and incorporated. The development of virtual, dynamic checklists/catalogues in which to capture, organize and easily update information about Canada’s biota represents an exciting challenge. There are many interesting models already available in Canada and globally that could be emulated or modified, although it is beyond the scope of this paper to review these. The two largest challenges are, firstly, for the community of data suppliers and users to form a consensus on what is needed (content, functionality, etc.) and, secondly, to find the resources to develop and sustain it long term. Without a broad base of support from a diversity of partners, the development and long-term maintenance of dynamic checklists will likely not be sustainable.

## Appendix I

**Diversity of Zygentoma in Canada**

Tomlin (1979) reported two species from Canada, both of which are non-native, cosmopolitan species that are domiciliary throughout much of the country and considered household pests: the silverfish, *Lepismasaccharina* L., and the firebrat, *Thermobiadomestica* (Packard). Tomlin (1979) predicted that another ten species might occur in the country and specifically mentioned two non-native, cosmopolitan species that were possibly established in domiciles in Canada: *Ctenolepiaimaurbana* Slasbaugh (now known as *Ctenolepismalongicaudata* Escherich, the grey silverfish) and *Ctenolepiaimaquadriseriata* Packard (now known as *Ctenolepismalineata* (Fabricius), the four-lined silverfish). These two species are now known to be established in Canada.

Sweetman and Kulash (1944) reported *C.lineata* (as *C.quadriseriata*) from “south-central Canada” but no specific localities were given. Numerous specimens of *C.lineata* from southern Ontario occur in the collections of the University of Guelph (S Paiero pers. comm.) and the Royal Ontario Museum (ROM), with the earliest collected in Toronto in 1929 (ROM; B Hubley, pers. comm). Thus, this species is confirmed from Canada but does not represent a new record as it was reported as early as 1944, but the record was missed by Tomlin (1979). A photograph of what appears to be *C.longicaudata* was taken in Burnaby, British Columbia in 2013 and submitted to iNaturalist (https://www.inaturalist.org/observations/2588314). This species is likely also to occur in southern Ontario and Quebec.

The basis for Tomlin’s (1979) estimate of a total fauna of 12 species in Canada is not known. Most of Canada can sustain only indoor populations of Zygentoma, but it is possible that southern parts of Ontario and British Columbia may have free-living species (MG Ricart pers. comm.). It seems unlikely that 12 species occur in Canada as only 18 species are known from North America and most of those have southern distributions (Arnett 2000). There is almost no information or data on which to base an informed estimate of Zygentoma faunal diversity in Canada as only two BINs are available (*Lepismasaccharina*: BOLD:ACB9678; *Thermobiadomestica*: BOLD:ACJ0722) and the distribution and identity of species in adjacent parts of the USA is poorly known. Given that ca. 37% of the terrestrial arthropods in North America occur in Canada (see above), based on the currently known North American Zygentoma fauna (18 spp.), seven species would be predicted to occur in Canada. Using a second estimation approach, if it can be assumed that Zygentoma are as well known as Archaeognatha in Canada (50% of species known), then the total Zygentoma fauna is expected to be eight species (i.e., four species remain undocumented).
